# Comparative transcriptomic analysis of functional and metabolic changes in mass-reared and wild *Anastrepha ludens* Loew (Diptera: Tephritidae)

**DOI:** 10.3389/finsc.2026.1913379

**Published:** 2026-08-17

**Authors:** Carlos Eduardo Aguilar-Castillo, Obdulia Lourdes Segura-León, Juan Cibrián-Tovar, Héctor González-Hernández, Juan Núñez-Farfán, Brenda Torres-Huerta

**Affiliations:** 1 Instituto de Fitosanidad, Colegio de Postgraduados, Texcoco, Estado de México, Mexico; 2 Departamento de Ecología Evolutiva, Instituto de Ecología, Universidad Nacional Autónoma de México, Ciudad de México, Mexico; 3 Departamento de Genética, Programa Operativo Moscas, Secretaría de Agricultura y Desarrollo Rural (SADER), Servicio Nacional de Sanidad, Inocuidad y Calidad Agroalimentaria (SENASICA), and Instituto Interamericano de Cooperación para la Agricultura (IICA), Metapa de Domínguez, Chiapas, Mexico

**Keywords:** chemosensory genes, differential gene expression, odorant-binding proteins, RNA-seq, sterile insect technique

## Abstract

**Introduction:**

Mass rearing for the Sterile Insect Technique (SIT) may induce transcriptomic changes that influence post-release performance. We compared head transcriptomes of two mass-reared *Anastrepha ludens* strains, Bisexual and Tap-7, with wild individuals associated with their preferred natural host, sweet orange.

**Methods:**

RNA-seq data were analyzed to assess transcriptomic differentiation, differential gene expression, Gene Ontology (GO) enrichment, Kyoto Encyclopedia of Genes and Genomes (KEGG) pathways, and chemosensory gene expression profiles among the three groups.

**Results:**

Principal component analysis clearly separated the mass-reared strains from wild host-associated individuals. More than 1,300 differentially expressed genes were identified per strain, with gene repression predominating and enrichment of functions related to translation, cellular energy metabolism, RNA processing, and metabolic regulation. Both mass-reared strains shared signatures of metabolic adjustment but also displayed strain-specific patterns. The Bisexual strain showed signatures associated with stress response, immunity, and mitochondrial quality control, whereas Tap-7 showed enrichment of pathways related to calcium regulation, secretion, detoxification, and folate metabolism. Both strains also exhibited reorganization of chemosensory gene expression, including conservation of receptors and co-receptors such as *Ir25a, Ir68b, Ir85a, Or74a*, and *SNMP1*, together with convergent downregulation of odorant-binding proteins, particularly *Obp56a*-like and members of the *Obp99* clade.

**Discussion:**

These results indicate that mass-reared *A. ludens* strains exhibit a distinct head transcriptomic state relative to wild host-associated flies. The shared and strain-specific expression patterns suggest that mass rearing may be associated with metabolic and sensory changes relevant to post-release performance. The identified sensory and metabolic markers provide candidates for future behavioral, electrophysiological, and physiological evaluation of fruit fly strains used in SIT programs.

## 1 Introduction

The Mexican fruit fly, *Anastrepha ludens* (Loew, 1873), is a polyphagous species native to northeastern Mexico that infests fruit crops of high economic importance, including citrus (*Citrus* spp.) and mango (*Mangifera indica*), as well as a broad range of secondary hosts belonging to families such as Myrtaceae and Rosaceae, among others (1, 2). In Mexico, *A. ludens* represents the most economically important fruit fly species and has been officially regulated under the National Fruit Fly Campaign since 1992 (3, 4). Its geographic distribution extends from the southern United States (Texas) to Panama (5).

Damage caused by *A. ludens* results from oviposition and larval development within the fruit, which facilitates secondary infections and leads to economic losses estimated between 10 and 15%, reaching up to 42% in some regions (6, 7). To mitigate these impacts, integrated pest management strategies have been implemented, including population monitoring, fruit sampling, host phenology analysis, cultural practices, selective baiting, augmentative biological control, regulatory measures, and the sterile insect technique (SIT). Among these, SIT is widely recognized for its effectiveness and its social, economic, and environmental benefits (8).

The sterile insect technique is a key strategy for controlling *A. ludens* in Mexico and relies on the sustained release of sterile males that compete with wild males for mating with wild females, thereby reducing the reproductive potential of the target population (9). Initially, the Moscafrut SIT program used bisexual strains, resulting in higher operational costs and reduced biological efficiency due to the simultaneous production and release of both males and females (10). The presence of females in mass-rearing systems decreases male yield per unit of diet and compromises operational efficiency, while their release in the field may reduce the effectiveness of the technique, as sterile females can cause damage through oviposition and indirectly interfere with mating dynamics by competing with wild females for sterile males (9).

These limitations led to the development of genetic sexing strains (GSS), which enable sex separation at early developmental stages, thereby improving production efficiency and reducing handling costs (11, 12). In this context, the Moscafrut program, in collaboration with the International Atomic Energy Agency (IAEA), developed the Tapachula-7 (Tap-7) GSS, which enables female removal by identifying black pupae, retaining only brown pupae (males) for irradiation and release. This represents a substantial advance in the operational optimization of SIT programs in Mexico (13).

However, intensive mass-rearing systems impose artificial selective pressures that may alter physiological, metabolic, and sensory processes, potentially affecting the biological quality and reproductive performance of released sterile males (14, 15). In particular, mass rearing and irradiation may affect functional processes related to chemosensory perception, energy metabolism, stress response, and reproduction, all of which are critical determinants of male sexual competitiveness under field conditions (16, 17). In this context, transcriptomic approaches based on RNA sequencing (RNA-seq) enable the comprehensive characterization of gene expression profiles under specific conditions (18) and provide a powerful framework for evaluating the functional and metabolic effects associated with mass-rearing systems beyond conventional quality-control indicators (14, 19).

Differential gene expression analysis in *A. ludens* enables the identification of molecular changes associated with chemosensory perception, energy metabolism, stress response, and reproduction (20). Studies in *Ceratitis capitata* and *Bactrocera dorsalis* have identified genes involved in volatile perception, plant compound digestion, oxidative stress, and fecundity, associated with adaptation to different host environments or experimental conditions (21, 22). However, transcriptomic information for *A. ludens* remains limited, focusing primarily on developmental stages and the characterization of odorant-binding proteins (OBPs) (23, 24).

The objective of this study was to identify gene expression patterns associated with the functional and metabolic effects of mass rearing in *A. ludens* and to characterize the major families of chemosensory genes, with particular emphasis on odorant-binding proteins (OBPs), through differential gene expression analyses of the Bisexual and Tapachula-7 strains in comparison with wild flies associated with their preferred host, orange. This approach sought to link molecular changes with processes related to adaptation, chemosensory perception, and reproductive performance relevant to SIT programs.

## 2 Materials and methods

### 2.1 Biological material collection

The biological material used in this study was obtained from the CP-CONACOFI-MOSCAFRUT projects conducted during 2019-2020. Wild *A. ludens* individuals originated from infested sweet orange (*Citrus sinensis*) fruits collected in Chiapas, Mexico. The fruits were maintained in rearing cages at the insectary of Colegio de Postgraduados (IFIT) under controlled environmental conditions of 28 ± 2 °C, a 12:12 h light photoperiod, and ambient humidity until adult emergence, after which males and females were separated. In addition, flies from the mass-reared Bisexual and Tapachula-7 (Tap-7) colonies were obtained from the Moscafrut facility in Metapa de Domínguez, Chiapas, Mexico. Only males from the three experimental groups were selected for subsequent analyses. Whole heads, including the antennae, compound eyes, mouthparts, and associated head tissues, were preserved in RNAlater™ Storage Solution (Thermo Fisher Scientific), without prior dissection or removal of specific sensory tissues. Samples were maintained at 4 °C for 24 h and subsequently stored at -20 °C until RNA extraction and processing at the Molecular Evolution Laboratory, Plant Health Program, Entomology and Acarology, Colegio de Postgraduados, Montecillo, Mexico.

### 2.2 RNA extraction, library preparation, and sequencing

Total RNA was extracted from pools of 20 adult male *A. ludens* heads per biological replicate for each of the three experimental groups. Samples were placed in microtubes containing RNAlater™ Storage Solution, following a completely randomized experimental design. Three biological replicates were included for each group: Bisexual (B-1, B-2, B-3), Tap-7 (T7-1, T7-2, T7-3), and Orange (N-1, N-2, N-3). Samples were sent to BGI (Hong Kong, China) for total RNA extraction, RNA-seq library preparation (cDNA), and sequencing. RNA quality and integrity were assessed using a Bioanalyzer 2100 (Agilent Technologies, USA), ensuring RNA integrity number (RIN) values ≥ 8. Sequencing was performed on the BGISEQ-500 platform, generating 100-bp paired-end reads.

### 2.3 Quality control, filtering, and alignment to the reference genome

Raw reads from each library were assessed using FastQC v0.10 (25), with standard quality metrics including per-base sequence quality, GC content, and adapter contamination. Reads were subsequently processed with FastP (26) to remove adapters and low-quality sequences. Filtered reads were aligned to the *A. ludens* reference genome (NCBI Assembly GCF_028408465.1, idAnaLude1.1, 2023) using Segemehl (27) with parameters appropriate for RNA-seq data.

### 2.4 Gene quantification and differential expression analysis

Aligned reads were processed with HTSeq-count (28) to quantify gene expression levels using the union counting mode. Raw counts were imported into RStudio and analyzed using the DESeq2 package (29) under a negative binomial distribution model. Genes with total counts ≤ 10 across the dataset were filtered out, and retained counts were normalized using the estimateSizeFactors() function. Wild males collected from infested sweet orange fruits were used as the reference group for contrasts against the Bisexual and Tap-7 strains. Global variation among samples was assessed using principal component analysis (PCA). Statistical significance values were adjusted using the Benjamini-Hochberg method to control the false discovery rate (FDR). Genes were considered differentially expressed (DEGs) if they met the thresholds of adjusted p-value (padj) ≤ 0.05 and absolute log2 fold change ≥ 2. DEG visualization was performed using volcano plots (EnhancedVolcano v1.20.0) and heatmaps (pheatmap v1.0.12).

### 2.5 Functional annotation and enrichment analysis

Functional annotation of DEGs was performed using a hierarchical strategy to maximize annotation coverage. First, genes with existing annotations in the *A. ludens* reference genome were retained. Genes lacking annotation were analyzed by homology-based searches against the *Drosophila melanogaster* reference proteome (UniProt proteome ID: UP000000803), using an e-value threshold of ≤ 1e−5 to assign putative functions based on sequence similarity (30). Genes without functional assignments were subsequently analyzed with InterProScan to identify conserved protein domains and families, thereby supporting functional inference (31). Remaining unassigned sequences were further annotated using eggNOG-mapper against the Insecta database (taxonomy ID: 50557), allowing the assignment of orthology-based functional categories and Gene Ontology (GO) terms (32). GO terms obtained from eggNOG-mapper outputs were classified into the three main ontological categories: Molecular Function (MF), Biological Process (BP), and Cellular Component (CC). In addition, the functional distribution of DEGs was evaluated using WEGO v2.0, grouping GO terms into BP, CC, and MF categories for the Bisexual and Tap-7 strains, compared with wild individuals (33). GO enrichment analysis was performed in RStudio using the GOseq package v1.54.0, which accounts for gene length bias inherent to RNA-seq data (34). GO terms with adjusted p-values (padj ≤ 0.05) were considered significantly enriched. Pathway enrichment analysis was conducted using clusterProfiler based on KEGG annotations, and pathways with adjusted p-values (padj ≤ 0.05) were considered significant (35, 36). Based on the functional annotations obtained, genes belonging to chemosensory families were identified, including odorant receptors (ORs), gustatory receptors (GRs), ionotropic receptors (IRs), sensory neuron membrane proteins (SNMPs), odorant-binding proteins (OBPs), and chemosensory proteins (CSPs). These genes were selected by searching for functional terms, conserved domains, and descriptors associated with each chemosensory family within the DEG dataset. Their expression patterns were subsequently analyzed using log2 fold change (log2FC) values and statistical significance (padj ≤ 0.05), according to the established differential expression criteria.

## 3 Results

### 3.1 Data processing, mapping, and principal component analysis

**Figure 1:**
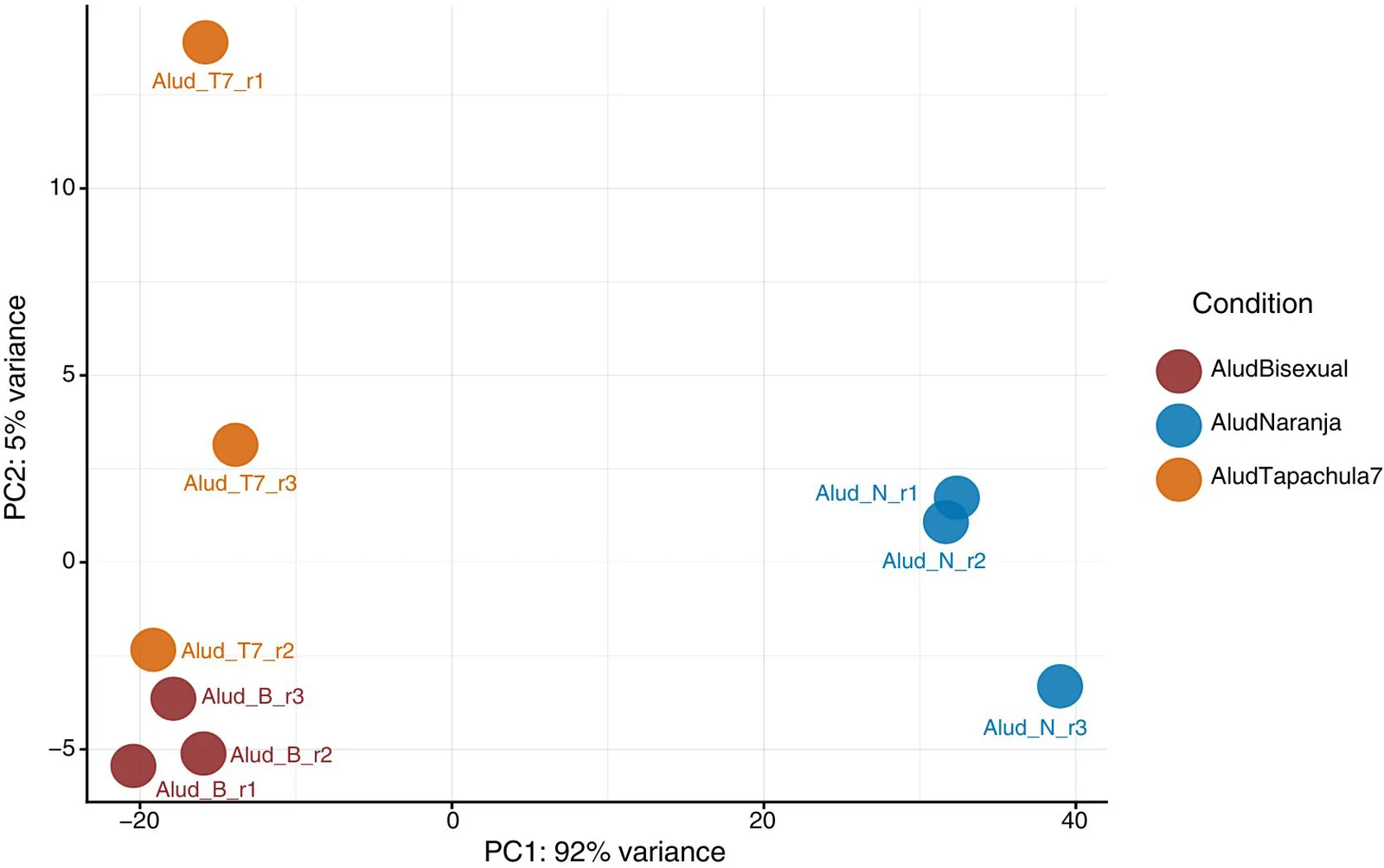
Principal component analysis (PCA) based on RNA-seq gene expression data in *A. ludens* from Bisexual and Tap-7 strains and wild individuals (from sweet orange).

Sequencing of the nine libraries on the BGISEQ-500 platform yielded an average of 46.64 million raw reads per library, with a mean read length of 100 bp. After quality filtering, an average of 42.97 million clean reads per library was retained, with a mean read length of 99 bp and quality values exceeding 89% of bases with Q ≥ 30. Alignment against the *A. ludens* reference genome (GCF_028408465.1) showed high mapping rates across all libraries, with an overall average of 93.99%. However, differences among conditions were observed, with higher mapping rates in wild individuals (96.97–97.82%) than in mass-reared strains, particularly Tap-7 (87.21–88.16%) (**Table 1**). Principal component analysis (PCA) revealed a clear separation between wild samples associated with sweet orange and the mass-reared Bisexual and Tap-7 strains (**Figure 1**). The first principal component (PC1) accounted for 92% of the total variance, indicating that most transcriptomic variation was associated with differences among the analyzed groups. The second principal component (PC2) explained an additional 5% of the variance and captured minor within-group variation, particularly among Tap-7 biological replicates. Together, PC1 and PC2 explained 97% of the total variance.

**Table 1:** Sequencing quality and mapping efficiency summary of RNA-seq libraries of *A. ludens* from Bisexual and Tap-7 strains and wild individuals (from orange fruits).

Condition	Clean reads (millions)	Clean bases (Gb)	GC content (%)	% ≥ Q30	Mapping (%)
B-1	43.30	4.33	39.24	91.9	95.01
B-2	44.19	4.42	38.42	91.86	95.95
B-3	42.85	4.28	38.99	91.77	95.23
T7-1	44.07	4.40	40.06	91.27	88.16
T7-2	48.73	4.87	40.02	89.14	87.75
T7-3	45.10	4.51	40.21	91.65	87.21
N-1	42.14	4.71	37.0	93.00	97.82
N-2	42.08	4.20	37.61	94.03	96.97
N-3	42.08	4.77	37.51	93.67	96.97

B, Bisexual; T7, Tapachula-7; N, wild individuals collected from sweet orange. % ≥ Q30: proportion of bases with Phred quality score ≥ 30, corresponding to an error probability ≤ 0.1% in base calling.

### 3.2 Differentially expressed genes and gene ontology functional classification

**Figure 2:**
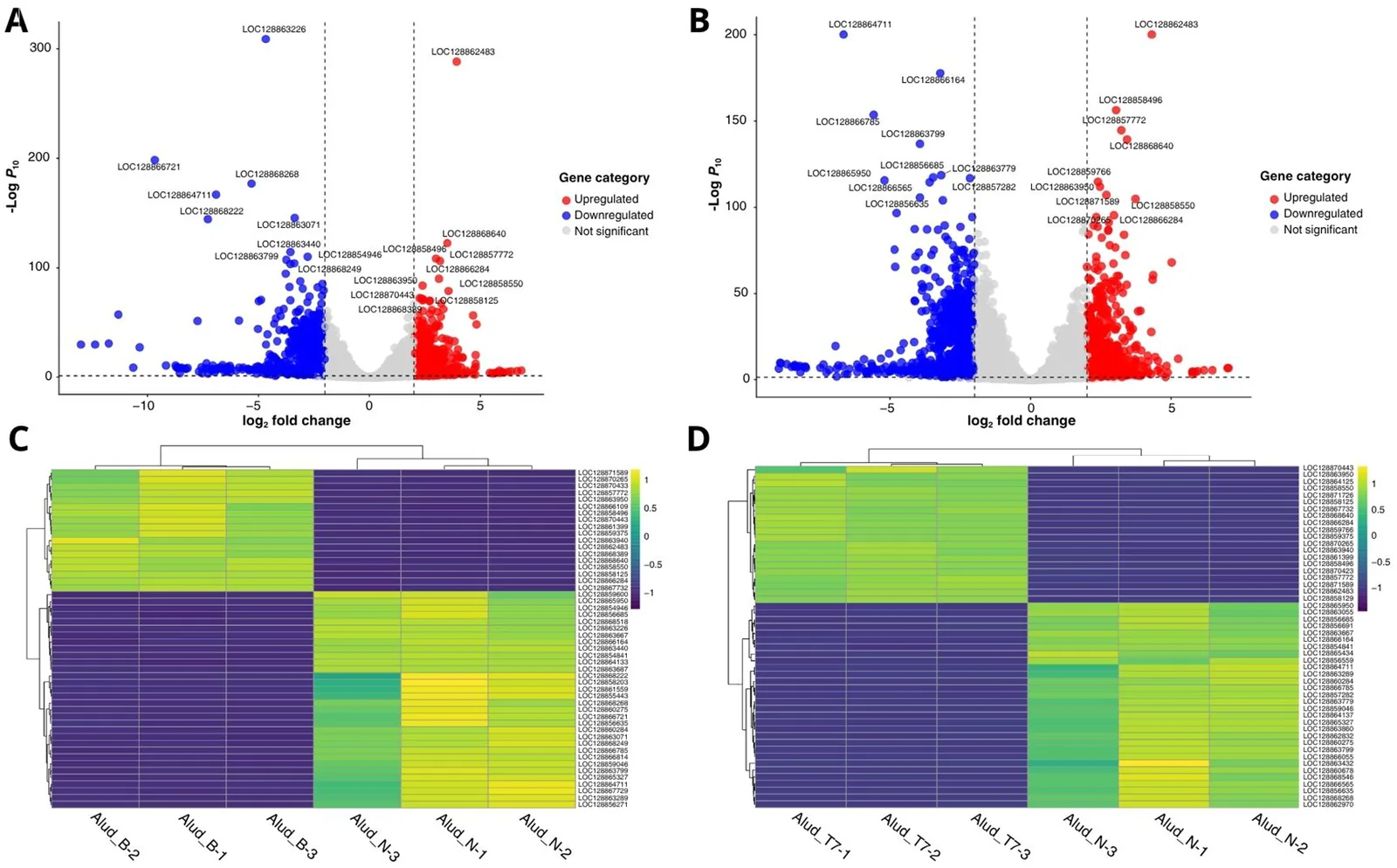
Volcano plots and heatmaps of differentially expressed genes (DEGs) in *A. ludens* for the contrasts Bisexual vs wild **(A, C)** and Tap-7 vs wild **(B, D)**, using wild males collected from sweet orange fruits as the reference group. In the volcano plots **(A, B)**, upregulated genes are shown in red, downregulated genes in blue, and non-significant genes in light gray. Dashed lines indicate the DEG thresholds: padj ≤ 0.05 and |log_2_FC| ≥ 2. In the heatmaps **(C, D)**, rows represent the 50 most statistically significant DEGs, selected by the lowest adjusted p-values, and columns represent biological replicates. Yellow indicates higher row-scaled relative expression, and blue indicates lower expression.

Differential expression analysis identified 1, 329 DEGs in the Bisexual strain and 1, 289 DEGs in the Tap-7 strain, both compared with wild individuals (**Figures 2A, B**). In both contrasts, downregulated genes were more abundant than upregulated genes. The Bisexual strain showed 745 downregulated and 584 upregulated genes, whereas Tap-7 showed 723 downregulated and 566 upregulated genes. A total of 680 DEGs were shared between both comparisons, representing approximately 51–52% of the DEGs in each contrast, while the remaining genes were strain-specific. This pattern suggests the presence of both common and strain-specific transcriptomic profiles. Volcano plots showed the distribution of DEGs according to log_2_FC and statistical significance (padj) (**Figures 2A, B**). Heatmaps based on the 50 most statistically significant DEGs, selected according to the lowest adjusted p-values, showed clear separation between mass-reared strains and wild individuals (**Figures 2C, D**), with some intra-group variability, particularly in Tap-7. Complete heatmaps including all DEGs for both comparisons are provided in **Supplementary Figure 1**.

**Figure 3:**
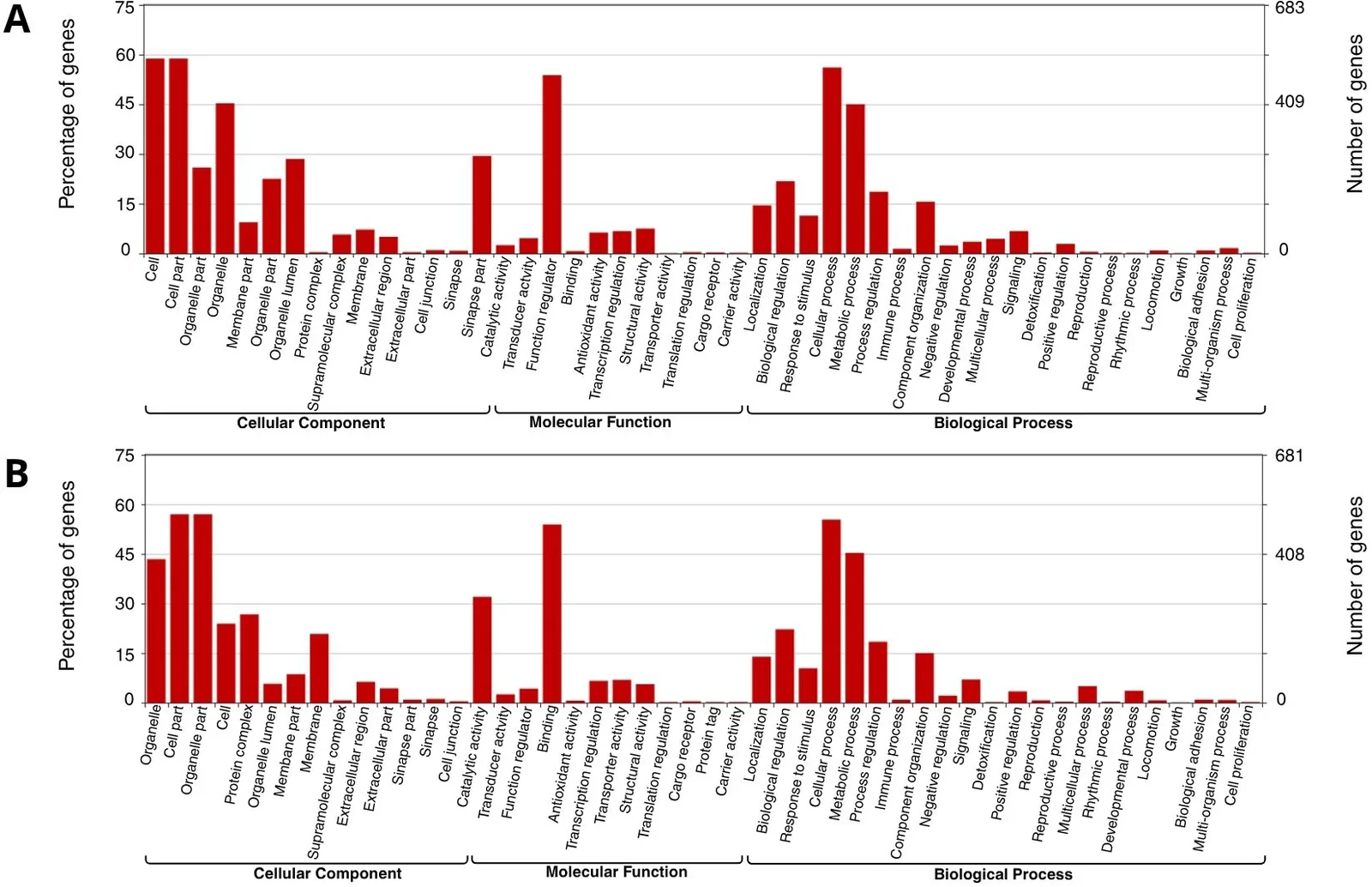
Gene Ontology (GO) functional classification using WEGO in the categories Biological Process (BP), Cellular Component (CC), and Molecular Function (MF) of differentially expressed genes (DEGs) in the contrasts Bisexual vs wild **(A)** and Tap-7 vs wild **(B)** in *A. ludens*, using wild individuals collected from sweet orange fruits as the reference group.

Functional classification using WEGO showed a consistent distribution of DEGs across the three main Gene Ontology (GO) categories: Biological Process (BP), Cellular Component (CC), and Molecular Function (MF) (**Figure 3**; **Table 2**). Within MF, terms related to binding and catalytic activity predominated; within CC, the most represented terms were associated with cellular components, organelles, and membranes; and within BP, cellular and metabolic processes were the most represented categories. Overall, these results indicate distinct gene expression profiles between mass-reared strains and wild individuals, likely associated with the combined effects of colonization history, rearing background, and genetic background.

**Table 2:** Number of differentially expressed genes (DEGs) annotated with WEGO by gene ontology (GO) category in the contrasts bisexual vs wild and Tap-7 vs wild in *A. ludens*.

Contrast	Annotated genes	GO Terms			Total
		BP	CC	MF	
Bisexual vs wild	911	655	666	731	2, 052
Tap-7 vs wild	908	644	635	743	2, 022

BP, Biological Process; CC, Cellular Component; MF, Molecular Function.

### 3.3 GO functional enrichment of differentially expressed genes

**Figure 4:**
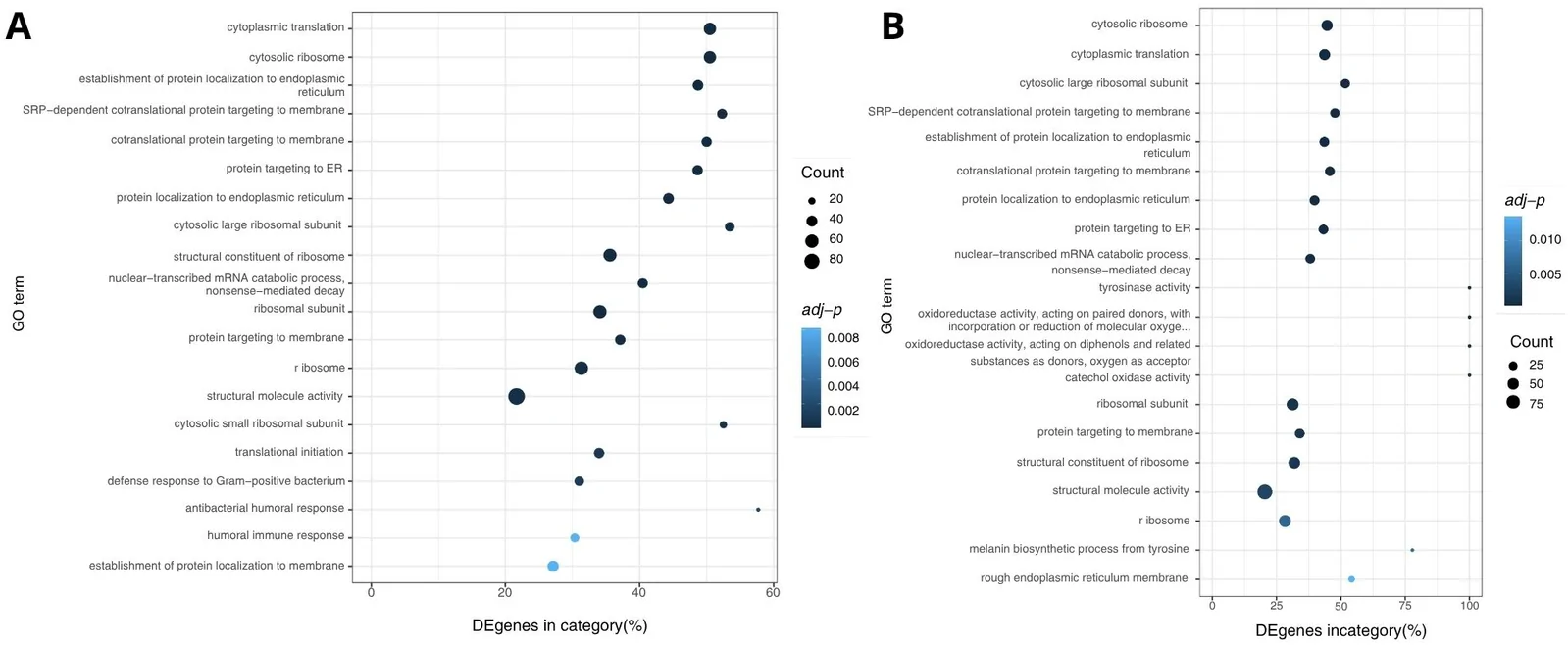
Gene Ontology (GO) term enrichment analysis using GOseq in the contrasts Bisexual vs wild **(A)** and Tap-7 vs wild **(B)** in *A. ludens*, using wild individuals collected from orange fruits as the reference group.

The GO enrichment analysis revealed differences in both the number and type of significantly enriched functional categories (padj ≤ 0.05) between the evaluated contrasts (**Figure 4**). In both comparisons, the terms cytoplasmic translation (GO:0002181) and cytosolic ribosome (GO:0022626) were among the most consistently enriched categories, along with other terms related to translation, ribosomal subunits, and protein targeting to membranes and the endoplasmic reticulum.

In the Bisexual vs wild comparison, 31 enriched GO terms were identified, of which 12 were not shared with Tap-7. These included terms such as humoral immune response (GO:0006959), antibacterial humoral response (GO:0019731), translation (GO:0006412), peptide biosynthetic process (GO:0043043), peptide metabolic process (GO:0006518), mRNA metabolic process (GO:0016071), cytosolic small ribosomal subunit (GO:0022627), extracellular region (GO:0005576), extracellular space (GO:0005615), ribonucleoprotein complex (GO:1990904), and large ribosomal subunit (GO:0015934) (**Figure 4A**; **Supplementary Table 1**). Overall, this profile indicates a stronger representation of processes associated with translation, peptide metabolism, and humoral immune response.

In the Tap-7 vs wild comparison, 27 enriched GO terms were identified, with seven unique terms to this contrast. These were mainly associated with melanin biosynthetic process (GO:0006583; GO:0042438), tyrosine metabolic process (GO:0006570), and regulation of striated muscle contraction (GO:0006942), as well as oxidative-related functions, including catechol oxidase activity (GO:0004097), tyrosinase activity (GO:0004503), and terms related to oxidoreductase activity (GO:0016682; GO:0016716) (**Figure 4B**; **Supplementary Table 1**). Collectively, this profile suggests a greater representation of melanogenesis, tyrosine metabolism, and oxidative processes in Tap-7.

In general, both contrasts shared a core functional signature associated with translation and ribosomal metabolism, while also displaying distinct enrichment patterns, indicating divergent functional trajectories between the Bisexual and Tap-7 strains relative to wild individuals.

### 3.4 KEGG pathway enrichment

**Figure 5:**
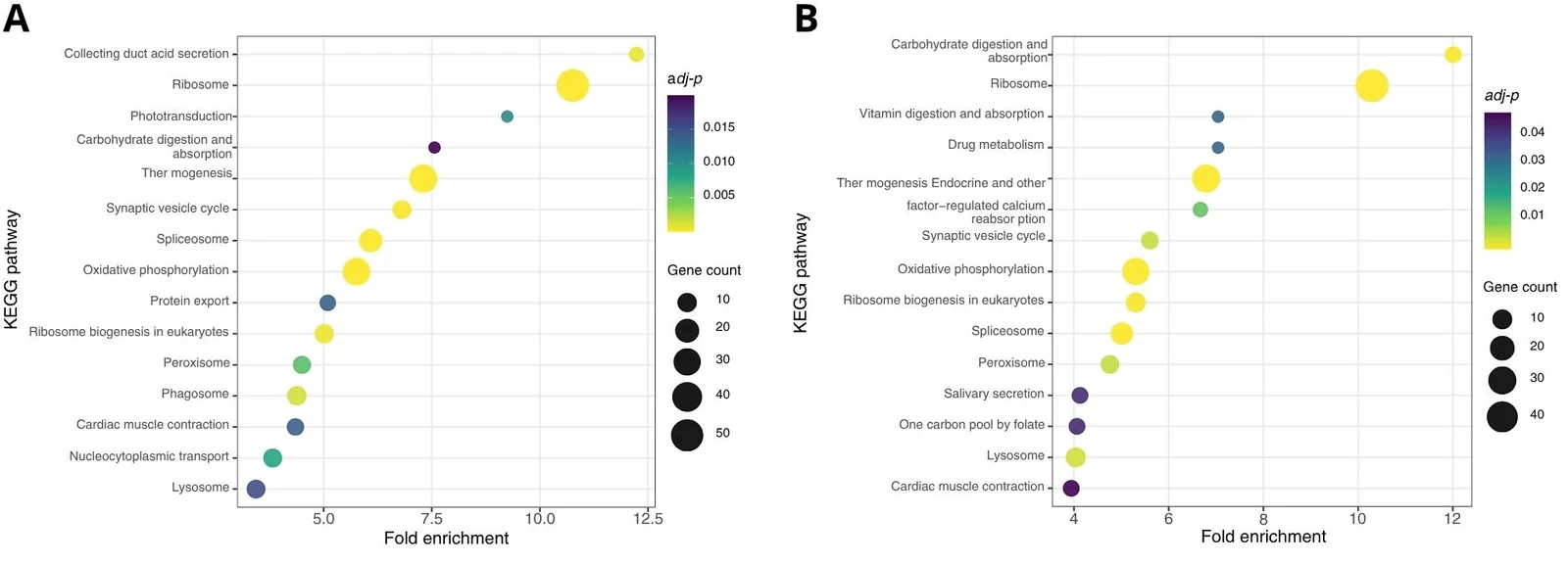
Significantly enriched KEGG pathways in *A. ludens* for the contrasts Bisexual vs. wild and Tap-7 vs. wild, using wild individuals collected from orange fruits as the reference group. **(A)** Top enriched KEGG pathways in the Bisexual vs. wild comparison. **(B)** Top enriched KEGG pathways in the Tap-7 vs. wild comparison. The x-axis represents fold enrichment, and the y-axis shows the enriched KEGG pathways. Dot size indicates the number of genes associated with each pathway, while dot color represents the adjusted p-value.

KEGG pathway enrichment analysis identified 20 significantly enriched pathways (padj ≤ 0.05) in the Bisexual vs wild comparison and 15 in the Tap-7 vs wild comparison (**Figure 5**, **Supplementary Table 2**). Among the shared pathways, Ribosome (ko03010) showed the highest statistical significance, with padj = 9.64 × 10^-37^ in Bisexual and padj = 6.38 × 10^-32^ in Tap-7, consistent with the GO enrichment results. Other shared pathways included Thermogenesis (ko04714), Oxidative phosphorylation (ko00190), and Spliceosome (ko03040), all of which are associated with energy metabolism and RNA processing (**Figure 5A**).

In the Bisexual vs wild comparison, several pathways were uniquely enriched, including Toll and Imd signaling pathway (ko04624), Protein export (ko03060), Mitophagy (ko04137), Carbohydrate digestion and absorption (ko04973), Axon regeneration (ko04361), Phototransduction (ko04745), and Collecting duct acid secretion (ko04966) (**Figure 5B**).

In contrast, the Tap-7 vs wild comparison showed unique enrichment of pathways such as Endocrine and other factor-regulated calcium reabsorption (ko04961), Drug metabolism (ko00982), Vitamin digestion and absorption (ko04977), Salivary secretion (ko04970), and One carbon pool by folate (ko00670) (**Figure 5B**).

Overall, both contrasts shared a core set of pathways related to protein synthesis and energy metabolism, while the distinct pathways suggest differences in physiological and metabolic processes between mass-reared strains and wild individuals.

### 3.5 Differential expression of chemosensory genes

Chemosensory gene families (ORs, GRs, IRs, SNMPs, CSPs, and OBPs) exhibited contrasting patterns of differential expression between the Bisexual vs wild and Tap-7 vs wild comparisons, both in the direction and magnitude of expression changes (see **Supplementary Table 3**).

In the Bisexual vs wild comparison, the highest number of differentially expressed chemosensory genes was observed, including both upregulated and downregulated genes. Among the upregulated genes, *Ir25a*, *Ir68b*, *Ir85a, Or74a-like*, and *SNMP1-like* were prominent. In contrast, downregulated genes included *Ir21a, Ir41a, Or7a-like, Gr8a-like, CSP OS-D-like*, and multiple OBPs (*Obp56a-like, Obp99b-like, Obp99c*, and *Obp99d)*.

In the Tap-7 vs wild comparison, the expression profile was characterized by the upregulation of *Ir25a, Ir68b, Ir85a, Ir92a, Or74a-like, Gr59e*, and *SNMP1-like*, along with the downregulation of several OBPs (*Obp56a-like, Obp99a, Obp99b-like, Obp99a-like, Obp99c*, and *Obp9d*) and *CSP OS-D-like.*

Overall, these results reveal distinct chemosensory expression profiles between both contrasts, particularly in the regulation of ionotropic receptors (IRs) and odorant-binding proteins (OBPs). Notably, while IRs tend to show consistent upregulation across both comparisons, OBPs exhibit a convergent pattern of downregulation, suggesting differential modulation between peripheral odorant detection (OBPs) and signal transduction mechanisms (IRs).

### 3.6 Differential expression of odorant-binding proteins

OBPs showed consistent downregulation in both mass-reared comparisons relative to wild individuals collected from orange fruits (**Table 3**), although differences were observed in the magnitude of expression change and the gene repertoire affected.

**Table 3:** Odorant-binding proteins (*Obp*s) with differential expression (|log_2_FC| ≥ 2; padj ≤ 0.05) in the contrasts bisexual vs wild and Tap-7 vs wild in *A. ludens*.

Contrast	Gene ID-NCBI	Gene name	log2FC	padj
Bisexual vs wild	LOC128866855	*Obp56a-like*	-11.24	6.59E-35
	LOC128866721	*Obp56a-like*	-9.66	9.74E-42
	LOC128855731	*Obp99d*	-2.6	1.47E-02
	LOC128871534	*Obp99c*	-2.53	2.53E-03
	LOC128860321	*Obp99b-like*	-2.36	1.74E-03
Tap-7 vs wild	LOC128866721	*Obp56a-like*	-5.81	6.16E-03
	LOC128855731	*Obp99d*	-2.34	2.58E-03
	LOC128871534	*Obp99c*	-2.3	7.02E-03
	LOC128854736	Obp99a*	-2.27	9.09E-07
	LOC128860321	*Obp99b-like*	-2.22	1.00E-02
	LOC128855732	Obp99a-like*	-2.06	3.44E-12
	LOC128860313	*Obp99b-like*	-2.01	4.93E-03

log_2_FC, log2 fold change; padj, adjusted p-value. Genes detected exclusively in one of the two contrasts.

In the Bisexual vs wild comparison, four OBPs were identified as differentially expressed, corresponding to five loci: *Obp56a-like, Obp99d, Obp99c*, and *Obp99b-like*. These genes showed variable levels of repression (log_2_FC ranging from -2.36 to -11.24). Notably, *Obp56a-like* exhibited the strongest downregulation, with log_2_FC values of -11.24 and -9.66 at two distinct loci (LOC128866855 and LOC128866721, respectively) (**Table 3**).

In the Tap-7 vs wild comparison, six OBPs were detected as differentially expressed, corresponding to seven loci: *Obp56a-like, Obp99d, Obp99c, Obp99a, Obp99b-like*, and *Obp99a-like*. In contrast, OBPs displayed a more uniform downregulation pattern (log_2_FC ranging from -2.01 to -5.81). *Obp99a* and *Obp99a-like* were exclusively detected in this comparison (**Table 3**). Overall, these results indicate a convergent downregulation of the OBP repertoire across both comparisons, with differences in the magnitude of repression and the specific loci involved.

## 4 Discussion

Transcriptomic tools have enabled a more precise characterization of molecular processes associated with physiology, environmental responses, and adaptation in agriculturally important insects (37, 38). In this study, global transcriptomic analysis showed a clear transcriptional divergence between adult male *A. ludens* from the mass-reared Bisexual and Tap-7 strains and wild individuals associated with their preferred host, sweet orange. This pattern suggests marked transcriptomic differentiation among the analyzed groups, which may reflect the combined effects of colony history, larval host or diet, and rearing background (39). In addition, the analysis focused on chemosensory genes and odorant-binding proteins (OBPs), which represent relevant candidate molecular markers because these components are involved in processes related to host localization, mate finding, and food searching under field conditions (23, 24). Overall, these results provide a framework for interpreting head-associated transcriptomic patterns and identifying candidate sensory and fitness-associated processes in *A. ludens* strains used in SIT programs.

### 4.1 Transcriptomic data quality and global differentiation among conditions

The libraries generated on the BGISEQ-500 platform showed high quality and consistency among biological replicates, with the percentage of bases ≥ Q30 ranging from 89.14% to 94.03%. Alignment of the reads against the *A. ludens* reference genome (GCF_028408465.1) showed an average mapping efficiency above 93%, comparable to or higher than that reported for other tephritids (40), such as *B. dorsa*lis and *C. capitata*, with mapping rates ranging from 77% to 81% (41). These results support the robustness of the dataset. Principal component analysis (PCA) revealed a clear separation between mass-reared strains and wild individuals, suggesting global differences in head gene expression associated with the analyzed groups. These differences may be related, at least in part, to larval host or diet, including development on an artificial diet based on corn protein rather than natural host substrates, as well as to colony history and rearing background. The overall clustering pattern supports the experimental consistency of the data, although differences in within-group dispersion were observed among strains (13, 42).

The greater dispersion among Tap-7 replicates in the PCA may reflect both sampling and biological factors. Tap-7 individuals were obtained from the same colony lot but collected on different days, whereas Bisexual samples were collected from the same lot on the same day. Thus, collection-day-associated variation may partly explain the higher within-group dispersion observed for Tap-7. Because the available quality-control metrics did not suggest an evident technical or sequencing-related batch effect, this pattern is more likely to reflect within-strain transcriptomic heterogeneity, potentially influenced by the genetic background and colony management history of Tap-7 as a genetic sexing strain. Overall, the technical quality, high mapping efficiency, and observed transcriptomic differentiation support the robustness of the differential expression and functional analyses. Nevertheless, the biological interpretation should consider the combined effects of mass-rearing status, larval diet, host origin, colony history, genetic background, and sampling day.

### 4.2 Transcriptional repression and GO functional enrichment: core metabolism and conserved cellular functions

The analysis revealed a similar predominance of downregulated genes in both mass-reared strains, suggesting that transcriptional repression was a shared pattern in the Bisexual and Tap-7 groups compared with wild individuals. Similar patterns have been reported in other tephritids, where reduced environmental complexity under laboratory or mass-rearing conditions has been associated with the downregulation of specialized functions (43). However, enrichment of terms related to translation and ribosome biogenesis in both strains indicates the maintenance of core biosynthetic machinery, consistent with studies in insects, including *C. capitata* under controlled conditions (10, 44). Differences in strain-specific enriched terms further suggest divergent head-associated transcriptional profiles between the Bisexual and Tap-7 strains. In the Bisexual strain, enrichment of categories associated with humoral immunity suggests modulation of immune-related transcripts in head tissue, which may be associated with the specific biological and rearing history of this strain. This pattern, described in other dipterans, may be coordinated with biosynthetic processes, mRNA metabolism, and vesicular transport, suggesting a possible relationship between translational capacity and post-transcriptional regulation (45, 46). Overall, these results indicate transcriptional reorganization in the Bisexual strain, potentially influenced by its colony history and genetic refreshing practices involving wild individuals (43).

In contrast, the Tap-7 strain showed enrichment of pathways associated with melanogenesis and tyrosine metabolism, indicating increased representation of oxidative-defense-related transcripts in the head transcriptome. These pathways have been linked to immune responses and stress-related processes in insects and may involve energetic costs or functional trade-offs under prolonged rearing conditions (47, 48). The enrichment of catechol oxidase, tyrosinase, and other oxidoreductases further supports a transcriptional profile associated with oxidative and defensive processes. Although melanization is an effective defense mechanism, sustained modulation of this pathway can generate reactive intermediates and increase energetic demands, potentially imposing physiological costs under prolonged rearing conditions (49, 50). In Tap-7, this pattern is consistent with a stronger redox-control-related transcriptional signature under its rearing background (15). In addition, enrichment of terms related to muscle contraction suggests differential representation of muscle-related transcripts in head tissue. Although these patterns may be relevant to traits such as flight performance and reproductive competitiveness, functional assays would be required to determine whether the observed transcriptional differences translate into measurable phenotypic effects (10). In *C. capitata*, modulation of muscle-related genes under mass-rearing conditions has been associated with variation in locomotor performance, sexual competitiveness, and longevity (10). In *A. ludens*, flight ability and reproductive success are sensitive to rearing and handling conditions (12), suggesting that these head-associated transcriptional signatures may identify candidate pathways relevant to SIT quality traits. Overall, although both strains shared global transcriptional repression and conservation of essential cellular functions, they differed in their enriched functional profiles: the Bisexual strain showed stronger enrichment of humoral immunity and biosynthetic-process-related terms, whereas Tap-7 showed enrichment of oxidative-pathway and muscle-related terms.

### 4.3 KEGG metabolic pathways: energy-related and functional signatures associated with mass-reared strains

KEGG analysis suggested the presence of energy-related and biosynthetic transcriptional signatures in *A. ludens* head transcriptomes. The shared enrichment of Ribosome, Spliceosome, Oxidative phosphorylation, and Thermogenesis in both mass-reared strains suggests coordinated modulation of pathways related to biosynthesis, RNA processing, and mitochondrial function. These patterns are consistent with transcriptional profiles associated with energetic demand reported in *A. ludens* and other tephritids, such as *B. dorsalis* (24, 51). Strain-specific pathways further indicated functional differentiation between the two mass-reared strains. In the Bisexual strain, enrichment of the Toll and Imd signaling pathways suggests modulation of immune-related transcripts in head tissue, possibly associated with microbiota-related changes under mass-rearing conditions, as previously described in tephritids (52). This pattern, together with the early enrichment of these pathways during development (24), suggests modulation of stress- and immunity-related modules that may be relevant to biological quality traits, such as survival and reproductive behavior. However, functional validation would be required to confirm these potential effects (15). The enrichment of Mitophagy further indicates increased representation of pathways related to mitochondrial quality control and energy redistribution, processes commonly associated with cellular homeostasis under stressful conditions. Although these transcriptional adjustments may support cellular maintenance, they may also reflect physiological trade-offs potentially affecting adult vigor, stress tolerance, and reproductive performance (53, 54). The enrichment of Carbohydrate digestion and absorption suggests a transcriptional profile related to energy acquisition, which may be consistent with the nutritional context of artificial diets designed to maximize productivity (55). In tephritids, early nutritional quality can have persistent effects on adult phenotype and stress tolerance, revealing trade-offs between early growth and later performance (54, 56). The enrichment of Protein export suggests high biosynthetic demand and protein trafficking toward the endoplasmic reticulum, a process associated with cellular stress responses and protein homeostasis. Similar pathway-level transcriptional patterns have also been reported in other tephritids, such as *B. minax* (57, 58). Neuronal pathways, including Phototransduction and Axon regeneration, suggest head-associated transcriptional signatures related to sensory maintenance and neuronal plasticity. In *A. ludens*, mating occurs in lekking systems mediated by visual, acoustic, and chemical signals; therefore, modulation of visual-system-related transcripts may identify candidate mechanisms potentially relevant to reproductive behavior and sexual competitiveness (6, 13). However, behavioral and electrophysiological assays would be required to determine whether these transcriptional patterns translate into functional differences. The role of vision as a key functional axis has also been documented in *B. minax* and *B. dorsalis* (59, 60). In addition, enrichment of Collecting duct acid secretion suggests modulation of ion transport and osmoregulation-related pathways mediated by V-ATPase, potentially associated with crowding, artificial diet, or other rearing-related conditions (61, 62). Overall, these results suggest transcriptional signatures related to sensory maintenance and homeostatic processes that may support laboratory performance, although their relationship with biological quality traits requires further functional validation.

In contrast to the Bisexual strain, Tap-7 showed enrichment of pathways associated with calcium regulation, including Endocrine and other factor-regulated calcium reabsorption; secretion-related pathways, such as Salivary secretion; detoxification-related pathways, such as Drug metabolism; micronutrient acquisition through Vitamin digestion and absorption; and folate metabolism through One carbon pool by folate and Antifolate resistance. Together, this profile suggests head-associated transcriptional signatures related to ion regulation, secretory activity, detoxification, and metabolic support in adults. Calcium-dependent signaling is central to neuronal excitability and glandular secretion, and enrichment of calcium- and secretion-related pathways may indicate modulation of processes potentially relevant to sensory orientation and reproductive fitness (63, 64). However, these interpretations should be considered hypotheses derived from transcriptomic data and require functional validation. The enrichment of Drug metabolism suggests basal detoxification-related transcriptional activity, potentially involving cytochrome P450s and conjugative enzymes. This pattern may be consistent with exposure to artificial diet components, microbial by-products, or other rearing-associated factors (65). In addition, enrichment of Vitamin digestion and absorption suggests metabolic support for the uptake of essential micronutrients, whereas the co-occurrence of One-carbon pool by folate and Antifolate resistance points to coordinated modulation of folate metabolism, a key process for biosynthesis and methylation (66, 67). These results suggest a transcriptional profile potentially associated with metabolic homeostasis and neurofunctional demands in Tap-7 adults under mass-rearing conditions, in agreement with previous evidence showing that diet can influence biological quality in *A. ludens* (67, 68). Together, these head transcriptomic signatures suggest two contrasting functional profiles in the mass-reared strains: the Bisexual strain showed enrichment of stress-, immunity-, and mitochondrial-maintenance-related pathways, whereas Tap-7 showed enrichment of pathways related to ion regulation, secretion, detoxification, and metabolic support. These profiles may identify candidate pathways relevant to biological quality in SIT programs, but their effects on adult performance and field competitiveness should be evaluated through behavioral, physiological, and electrophysiological assays.

### 4.4 Alterations in the chemosensory repertoire under mass rearing and host adaptation

Differences in the expression of chemosensory genes, including ORs, GRs, IRs, SNMPs, CSPs, and OBPs, between the Bisexual and Tap-7 strains and wild host-associated individuals suggest a reorganization of chemosensory-related transcriptional profiles. This pattern is consistent with transcriptional plasticity associated with the biological and rearing history of the analyzed strains, rather than evidence of mass-rearing effects alone. Similar transcriptional changes have been documented in insects maintained under prolonged artificial conditions, where homogeneous diets and controlled environments may reduce the need for complex host-cue detection and favor alternative chemosensory expression profiles (69). The most consistent pattern in both strains, compared with wild individuals, was the coordinated downregulation of multiple OBPs, including *Obp56a-like* and the *Obp99* cluster, as well as *CSP OS-D-like*. In *A. ludens*, these families have been extensively characterized and represent key components of the olfactory system (21), with proteomic evidence confirming the abundance of OBPs such as *Obp99b* in antennal tissues (70). Similar patterns observed in *C. capitata* suggest that these peripheral proteins may be sensitive to environmental and rearing-related conditions (22). Overall, the downregulation of OBPs and CSPs suggests reduced expression of peripheral chemosensory carrier proteins involved in the modulation of complex chemical signals. This pattern is consistent with possible sensory-related adjustments associated with artificial diets, homogeneous environments, and colony history (71, 72). Such changes may reflect reduced fine-scale chemical discrimination capacity, potentially influencing orientation, host searching, and responses to environmental cues; however, behavioral or electrophysiological assays would be required to confirm these functional effects (52, 60). In parallel, changes in ORs, including the upregulation of *Or74a-like* in both strains and the downregulation of *Or7a-like* in the Bisexual strain, suggest strain-associated differences in the expression of receptors involved in volatile compound detection. These receptors have been associated with the detection of aggregation-related compounds such as 1-nonanol/6-oxo-1-nonanol and 9-tricosene, respectively, and are part of the chemosensory repertoire of *A. ludens* (23, 73). The upregulation of *Or74a-like* may reflect increased expression of receptors related to cues commonly encountered under rearing conditions, whereas downregulation of *Or7a-like* may indicate reduced expression of receptors associated with aggregation-related signals (74). These OR expression patterns identify candidate genes potentially relevant to orientation and mating-site localization in sterile males used in SIT programs, but their behavioral relevance remains to be experimentally validated. In both mass-reared strains, the upregulation of *Ir25a, Ir68b*, and *Ir85a* suggests conservation, and possibly increased expression, of a generalist environmental sensory core. This pattern may be consistent with the need to monitor microenvironmental conditions and chemical cues associated with artificial diets or rearing environments. In *A. ludens*, these IRs have been linked to functions such as thermo- and hygrosensation and the detection of compounds derived from fermentation processes (23, 75, 76). However, the expression pattern differed between strains. In the Bisexual strain, downregulation of Ir21a and Ir41a suggests reduced expression of channels associated with thermo-orientation and amine detection, whereas upregulation of *Ir92a* in Tap-7 may indicate increased expression of receptors associated with nitrogen-containing compounds typical of fermentative environments (77, 78). These results suggest that the analyzed mass-reared strains retain expression of general sensory modules potentially relevant to artificial rearing environments, whereas components involved in fine-scale orientation differ between strains. These patterns may have implications for host-cue responses and post-release behavior, but functional validation is required. Changes in GRs, including the upregulation of *Gr59e-like* in Tap-7 and the modulation of *Gr8a-like* in the Bisexual strain, suggest strain-associated differences in gustatory receptor expression that may be related to the artificial feeding regime. Both genes correspond to orthologs previously identified in *A. ludens* (23) and have been linked in dipterans to the detection of aversive compounds and the regulation of feeding decisions (79, 80). Differential expression of these GRs (log_2_FC ≈ 2.8) suggests a possible reconfiguration of gustatory-related transcriptional profiles, potentially associated with food acceptance thresholds and energy balance under rearing conditions. These changes may identify candidate mechanisms related to feeding behavior and energetic resource acquisition, with possible relevance to flight and sexual competitiveness after release; however, these potential effects require direct behavioral and physiological assessment. Likewise, the upregulation of *SNMP1-like* in both strains suggests the conservation of key components of olfactory and pheromonal signal transduction. In insects, *SNMP1* is part of the pheromone receptor complex and is essential for efficient detection of chemical signals (81, 82). It has also been reported in antennal tissues of *A. ludens, B. dorsalis*, and *C. capitata* (23, 83, 84). The maintenance of *SNMP1-like* expression, together with the reduction of OBPs, suggests a possible compensatory transcriptional pattern in which components involved in signal transduction may be preserved while peripheral carrier proteins involved in complex host-odor processing are reduced. This pattern is consistent with chemosensory transcriptional reorganization associated with artificial mass-rearing environments (85). Differences between the *A. ludens* strains suggest divergent chemosensory transcriptional profiles. The Bisexual strain showed a greater number of modulated chemosensory genes and mixed expression directions, suggesting stronger divergence from the wild host-associated expression profile. In contrast, Tap-7 displayed a more conserved profile, characterized by retention of the IR/OR/SNMP core and consistent downregulation of peripheral proteins, including OBPs and CSPs. This divergence is consistent with observations in SIT systems, where differences in colonization history, rearing background, and strain management can yield alternative transcriptomic outcomes that may be relevant to biological quality (71).

### 4.5 OBP downregulation: ecological and behavioral implications

OBPs showed consistent downregulation in both mass-reared strains compared with wild individuals, suggesting a convergent reduction in the expression of genes associated with the peripheral component of odorant detection. In tephritids, these proteins are closely linked to host localization and sexual recognition and are predominantly expressed in antennal tissues (22, 23). In the Bisexual strain, four OBPs were downregulated (*Obp56a-like, Obp99d, Obp99c*, and *Obp99b-like*), with particularly strong repression of *Obp56a-like* at two loci (log_2_FC = -11.24 and -9.66). In contrast, Tap-7 showed six downregulated OBPs, with a more uniform decrease (log_2_FC ≈ -2 to -5.8) and the inclusion of *Obp99a*/*Obp99a-like* as strain-specific genes. Although both strains showed reduced OBP expression relative to wild individuals, they differed in their transcriptional profiles: Tap-7 showed a broader reduction across OBP genes, whereas the Bisexual strain exhibited stronger repression of specific OBP loci. These differences may reflect variation in colonization history, rearing background, or strain-specific transcriptional plasticity. *Obp56a-like* and members of the *Obp99* clade (*Obp99a/b/c/d*) correspond to proteins broadly expressed in olfactory tissues of dipterans and associated with the detection and discrimination of chemical cues (23, 85, 86). In this context, the marked repression of *Obp56a-like*, together with the coordinated reduction of multiple *Obp99* genes, suggests reduced expression of peripheral odorant-binding components that may participate in odorant filtering and fine-scale odor discrimination. This pattern is consistent with possible chemosensory transcriptional remodeling associated with artificial rearing environments, where the chemical spectrum is more restricted than in natural host environments. However, whether these changes translate into reduced host orientation or altered behavioral responses after release remains to be tested through behavioral and electrophysiological assays. This interpretation is consistent with previous reports of reduced reproductive performance in mass-reared males and differences in olfactory sensitivity between wild and laboratory populations of tephritids (73, 87, 88). Nevertheless, because olfactory function also depends on membrane receptors, co-receptors, neural processing, and behavioral context, mass-reared strains may partially retain responses to key cues through other sensory modules, even if the expression of peripheral odorant-binding components is reduced.

### 4.6 Implications for the sterile insect technique

From an applied perspective, the transcriptomic patterns observed in this study, including OBP downregulation, differential expression of OR/IR receptors, conservation of central chemosensory components such as *SNMP1* and *Ir25a*, and enrichment of pathways associated with energy metabolism, secretion, detoxification, and neuronal signaling, may be relevant for SIT programs. The sexual competitiveness of released males depends on the integration of sensory perception, physiological condition, and metabolic homeostasis. In this context, reduced expression of specialized peripheral chemosensory genes, together with modulation of bioenergetic and neuronal signaling pathways, suggests a transcriptional profile potentially associated with laboratory rearing environments. Although this profile may be compatible with productivity under laboratory conditions, it could also be associated with changes in the ability to detect or respond to ecological cues after release. These transcriptional signatures provide candidate molecular mechanisms that may help explain previously reported differences in behavioral performance and reproductive success between mass-reared and wild insects, even in the absence of evident morphological changes. However, direct behavioral, physiological, and electrophysiological validation will be required to determine the functional consequences of these expression patterns. Overall, our results suggest that biological quality in SIT programs may depend not only on physical parameters but also on the functional integrity of sensory and metabolic networks. This highlights the potential value of incorporating molecular markers based on GO/KEGG analyses and gene expression profiles as complementary tools for strain evaluation and optimization.

## 5 Study limitations and future directions

Several limitations should be considered when interpreting these results. The comparison between wild and mass-reared groups involves multiple confounding factors, including larval host or diet, colony history, rearing background, genetic background, and sampling conditions. Therefore, the transcriptomic differences observed here should be interpreted as correlative patterns associated with the combined biological and rearing histories of the analyzed groups, rather than as causal effects exclusively attributable to mass rearing. In addition, because RNA was extracted only from adult male heads, GO and KEGG categories related to immunity, muscle contraction, stress response, mitochondrial function, secretion, detoxification, and metabolism should be considered head-associated transcriptional signatures rather than direct evidence of whole-body physiological changes. Finally, because RNA-seq results were not independently validated by RT-qPCR, behavioral assays, electrophysiological assays, or physiological assays, the identified genes and pathways should be considered candidate molecular markers requiring further validation.

## 6 Conclusions

The transcriptomic analysis revealed marked differentiation between mass-reared *A. ludens* strains used in SIT programs and wild individuals associated with sweet orange. These differences were characterized by a predominance of gene repression and enrichment of processes associated with translation, mitochondrial bioenergetics, RNA processing, and chemosensory function. Rather than indicating effects exclusively caused by mass rearing, these patterns should be interpreted as transcriptomic signatures associated with the combined biological and rearing histories of the analyzed groups, including colony history, larval host or diet, and rearing background. Both strains showed shared transcriptional features but also divergent functional profiles. The Bisexual strain displayed head-associated signatures related to stress response, immunity, and mitochondrial quality control, whereas Tap-7 showed enrichment of pathways related to ion regulation, secretion, detoxification, and folate metabolism. Notably, both strains exhibited chemosensory transcriptional remodeling, characterized by the conservation of central receptors and co-receptors together with the convergent downregulation of peripheral OBPs. This pattern suggests that basic sensory-related components may be maintained, whereas genes potentially involved in fine-scale host-cue discrimination show reduced expression. However, the behavioral consequences of these expression patterns remain to be tested through olfactory, electrophysiological, and field-performance assays. Overall, these results identify candidate neuro-metabolic and chemosensory markers potentially relevant to biological quality in SIT programs. Incorporating molecular markers derived from GO/KEGG analyses and chemosensory gene expression profiles may complement conventional quality-control parameters for strain evaluation and optimization.

## Data Availability

The datasets presented in this study can be found in online repositories. The names of the repository/repositories and accession number(s) can be found in the article/**Supplementary Material**.

## Supplementary material

Supplementary Figure 1 Complete heatmaps of all differentially expressed genes (DEGs) identified in A. ludens for the contrasts Bisexual vs wild **(A)** and Tap-7 vs wild **(B)**, using wild males collected from sweet orange fruits as the reference group. Rows represent all DEGs meeting the thresholds padj ≤ 0.05 and |log2FC| ≥ 2, and columns represent biological replicates. The color scale represents rowscaled relative expression levels, with yellow indicating higher relative expression and blue/purple indicating lower relative expression.
