# Supplementary material for: Comparative transcriptomic analysis of functional and metabolic changes in mass-reared and wild Anastrepha ludens Loew (Diptera: Tephritidae)
Source: Front Insect Sci. 2026 Aug 17;6:1913379. doi: 10.3389/finsc.2026.1913379 (PMC13527029; doi:10.3389/finsc.2026.1913379)
Supplement: Supplementary file 1 [file Table1.docx]

Table S1. Gene Ontology (GO) enrichment analysis of differentially expressed genes identified in the Bisexual and Tap-7 strains relative to wild individuals.

| **Condition** | **Category** | **DEGs** | **EG** | **Term** | **Ontology** | **padj** |
| --- | --- | --- | --- | --- | --- | --- |
| Bisexual | GO:0002181 | 52 | 103 | cytoplasmic translation | BP | 7.94E-12 |
|  | GO:0072599 | 38 | 78 | establishment of protein localization to endoplasmic reticulum | BP | 1.14E-07 |
|  | GO:0006614 | 34 | 65 | SRP-dependent cotranslational protein targeting to membrane | BP | 1.77E-07 |
|  | GO:0006613 | 35 | 70 | cotranslational protein targeting to membrane | BP | 2.26E-07 |
|  | GO:0070972 | 39 | 88 | protein localization to endoplasmic reticulum | BP | 3.38E-07 |
|  | GO:0045047 | 36 | 74 | protein targeting to ER | BP | 3.38E-07 |
|  | GO:0000184 | 34 | 84 | nuclear-transcribed mRNA catabolic process, nonsense-mediated decay | BP | 3.09E-05 |
|  | GO:0006612 | 36 | 97 | protein targeting to membrane | BP | 1.03E-04 |
|  | GO:0006413 | 36 | 106 | translational initiation | BP | 9.77E-04 |
|  | GO:0050830 | 31 | 100 | defense response to Gram-positive bacterium | BP | 1.11E-03 |
|  | GO:0019731 | 15 | 26 | antibacterial humoral response* | BP | 1.72E-03 |
|  | GO:0006959 | 27 | 89 | humoral immune response* | BP | 8.17E-03 |
|  | GO:0090150 | 42 | 155 | establishment of protein localization to membrane | BP | 8.17E-03 |
|  | GO:0043043 | 79 | 331 | peptide biosynthetic process* | BP | 8.17E-03 |
|  | GO:0006412 | 77 | 317 | translation* | BP | 8.17E-03 |
|  | GO:0006518 | 89 | 402 | peptide metabolic process* | BP | 9.79E-03 |
|  | GO:0110077 | 5 | 5 | vesicle-mediated intercellular transport* | BP | 3.04E-02 |
|  | GO:0016071 | 95 | 500 | mRNA metabolic process* | BP | 3.04E-02 |
|  | GO:0060314 | 5 | 7 | regulation of ryanodine-sensitive calcium-release channel activity | BP | 4.00E-02 |
|  | GO:0022626 | 52 | 103 | cytosolic ribosome | CC | 9.68E-12 |
|  | GO:0022625 | 31 | 58 | cytosolic large ribosomal subunit | CC | 3.87E-07 |
|  | GO:0044391 | 60 | 176 | ribosomal subunit | CC | 5.70E-05 |
|  | GO:0005840 | 62 | 198 | ribosome | CC | 2.88E-04 |
|  | GO:0022627 | 21 | 40 | cytosolic small ribosomal subunit* | CC | 4.07E-04 |
|  | GO:0030867 | 13 | 24 | rough endoplasmic reticulum membrane | CC | 1.99E-02 |
|  | GO:0005576 | 140 | 915 | extracellular region* | CC | 2.46E-02 |
|  | GO:0005615 | 84 | 490 | extracellular space* | CC | 3.34E-02 |
|  | GO:1990904 | 125 | 662 | ribonucleoprotein complex* | CC | 3.78E-02 |
|  | GO:0015934 | 36 | 110 | large ribosomal subunit* | CC | 3.78E-02 |
|  | GO:0003735 | 58 | 163 | structural constituent of ribosome | MF | 3.09E-05 |
|  | GO:0005198 | 97 | 448 | structural molecule activity | MF | 2.89E-04 |
| Tap-7 | GO:0002181 | 45 | 103 | cytoplasmic translation | BP | 5.63E-08 |
|  | GO:0006614 | 31 | 65 | SRP-dependent cotranslational protein targeting to membrane | BP | 1.06E-05 |
|  | GO:0072599 | 34 | 78 | establishment of protein localization to endoplasmic reticulum | BP | 1.11E-05 |
|  | GO:0006613 | 32 | 70 | cotranslational protein targeting to membrane | BP | 1.11E-05 |
|  | GO:0070972 | 35 | 88 | protein localization to endoplasmic reticulum | BP | 3.41E-05 |
|  | GO:0045047 | 32 | 74 | protein targeting to ER | BP | 4.44E-05 |
|  | GO:0000184 | 32 | 84 | nuclear-transcribed mRNA catabolic process, nonsense-mediated decay | BP | 1.82E-04 |
|  | GO:0006612 | 33 | 97 | protein targeting to membrane | BP | 1.10E-03 |
|  | GO:0006583 | 7 | 9 | melanin biosynthetic process from tyrosine* | BP | 7.17E-03 |
|  | GO:0006570 | 10 | 21 | tyrosine metabolic process* | BP | 1.75E-02 |
|  | GO:0006413 | 32 | 106 | translational initiation | BP | 1.82E-02 |
|  | GO:0006942 | 11 | 34 | regulation of striated muscle contraction* | BP | 1.82E-02 |
|  | GO:0042438 | 11 | 27 | melanin biosynthetic process* | BP | 1.87E-02 |
|  | GO:0050830 | 27 | 99 | defense response to Gram-positive bacterium | BP | 2.28E-02 |
|  | GO:0090150 | 39 | 155 | establishment of protein localization to membrane | BP | 3.73E-02 |
|  | GO:0060314 | 5 | 7 | regulation of ryanodine-sensitive calcium-release channel activity | BP | 4.09E-02 |
|  | GO:0022626 | 46 | 103 | cytosolic ribosome | CC | 5.08E-08 |
|  | GO:0022625 | 30 | 58 | cytosolic large ribosomal subunit | CC | 2.33E-06 |
|  | GO:0044391 | 55 | 176 | ribosomal subunit | CC | 8.88E-04 |
|  | GO:0005840 | 56 | 198 | ribosome | CC | 6.50E-03 |
|  | GO:0030867 | 13 | 24 | rough endoplasmic reticulum membrane | CC | 1.37E-02 |
|  | GO:0004097 | 7 | 7 | catechol oxidase activity* | MF | 6.06E-04 |
|  | GO:0004503 | 7 | 7 | tyrosinase activity* | MF | 6.06E-04 |
|  | GO:0016682 | 7 | 7 | oxidoreductase activity, acting on diphenols and related substances as donors, oxygen as acceptor* | MF | 6.06E-04 |
|  | GO:0016716 | 7 | 7 | oxidoreductase activity, acting on paired donors, with incorporation or reduction of molecular oxygen, another compound as one donor, and incorporation of one atom of oxygen* | MF | 6.06E-04 |
|  | GO:0003735 | 52 | 163 | structural constituent of ribosome | MF | 1.22E-03 |
|  | GO:0005198 | 92 | 450 | structural molecule activity | MF | 2.69E-03 |

DEGs: differentially expressed genes. EG: enriched genes. *: GO terms exclusively enriched in the Bisexual and Tap-7 strains relative to wild individuals.
